# Conductivity Behaviour under Pressure of Copper Micro-Additive/Polyurethane Composites (Experiment and Modelling)

**DOI:** 10.3390/polym14071287

**Published:** 2022-03-23

**Authors:** Saeid Mehvari, Yolanda Sanchez-Vicente, Sergio González, Khalid Lafdi

**Affiliations:** Department of Mechanical & Construction Engineering, Faculty of Engineering & Environment, Northumbria University Newcastle, Newcastle upon Tyne NE1 8ST, UK; saeid.mehvari@northumbria.ac.uk (S.M.); sergio.sanchez@northumbria.ac.uk (S.G.); khalid.lafdi@northumbria.ac.uk (K.L.)

**Keywords:** electrical conductive composite, micro-copper, polyurethane, percolation threshold, RVE model, metal filler, numerical simulation

## Abstract

In this study, micro-size copper particles (less than 25 μm) were incorporated into polyurethane (PU) using a solution mixing method and spin-coating technique to fabricate composite films in concentrations from 0.5 to 20 vol.%. The conductivity behaviour of these composites under pressure was studied experimentally and numerically. The conductivity measurements were performed in-plane and through-thickness under pressure. It was found that changes in conductivity only occurred in the z-direction under an applied pressure from 1 to 20 kPa. The results showed that pressure could induce conductivity up to about 7.2 × 10^−1^ S∙m^−1^ for composites with a Cu concentration higher than 2.6 vol.%. It seems that applied pressure reduced the thickness of the polymer film, decreasing the distance between copper particles and promoting the formation of a conductive network, thus making the material conductive. A semi-analytical model that can accurately provide the percolation threshold (PT) concentration was used to fit the experimental conductivity. The PT concentrations for PU-Cu composite ranged from 7.1 vol.% to 1.4 vol.% and decreased with the rise in pressure. This is known as a pressure-induced percolation transition phenomenon (PIPT). Finally, the finite element method based on the representative volume element model (FE-RVE) simulation technique was used to predict the conductivity behaviour. This numerical simulation provided a good description of the experimental conductivity after the PT and correctly predicted the PT concentration. This study shows that FE-RVE could be used to effectively simulate the influence of pressure on the electrical properties of a polymer–metal composite, reducing the need for costly and time-consuming experiments.

## 1. Introduction

Polymers were thought of as electrical insulators before the discovery of intrinsically conductive polymers (ICP). ICPs are organic polymers containing conjugated single and double bonds in the long-chain (backbone) to carry current. Polyaniline, polyazulene, polypyrrole, polyacetylene, polythiophene, polyaminonaphthalenes, and their derivatives are the typical ones [1]. However, ICPs have been shown to act as insulators or semiconductors in their natural state. They show metallic conductivity only when they are doped with an appropriate dopant or mixed into a composite with other materials [2]. Furthermore, they have some limitations in biomedical applications due to their cytotoxic nature [3]. Another method of transforming insulating polymers into conductive polymers is through the dispersion of conductive fillers in the polymer matrix. These materials are known as electrically conductive polymer composites (ECPCs). Therefore, ECPCs are composite materials that mainly contain two components: an insulating matrix (polymer) and a conductive filler [4]. Carbon nanotubes (CNT) [5], carbon fiber [6], carbon black [7], graphite [8], graphene [9], metal particles (nano- and microparticles) [10], metal nanowires [11], and/or ICPs [1], are usually used as a conductive fillers to produce ECPCs. ECPCs combine some of the properties of polymers, such as low density, ease of processing, corrosion resistance, and low cost, while also exhibiting favourable electrical properties from the filler network. Therefore, they have been used in numerous technological applications such as sensors [12], supercapacitors [13], rechargeable batteries [14], anti-corrosion films [15], biomedical devices [3], organic solar cells [16], electrochromic [17], and smart fabrics [18].

Metal nanoparticles and nanowires are common fillers added into the polymer to enhance their electrical properties [19]. Among them, silver nanoparticles and nanowires are the most commonly used because of their high electrical conductivity (6.30 × 10^7^ S∙m^−1^) [20]. Copper nanoparticles and nanowires might be attractive alternatives to replace silver as a conductive nanomaterial. Copper also possesses high electrical conductivity (5.96 × 10^7^ S∙m^−1^) [20], but it is more abundant and cost-effective than silver [19]. Although nanocomposites offer numerous advantages, they also have certain drawbacks. For example, the cost of synthesizing nanomaterials remains expensive, and scaling up nanoparticle production to an industrial level is still a challenging task [21]. Furthermore, nanoparticles can penetrate within the cells and affect the existing chemical transformation routes in the cells. The fact is that the impact of these nanomaterials on human health and the environment is still unknown [22]. As a result, using metal microparticles to create polymer composites with electrical properties might be the safest alternative. On the other hand, polymer matrix selection is also a critical aspect in the fabrication and application of ESPCs. Elastomers with excellent flexibility and stretchability are commonly utilized as a matrix, particularly for sensor and smart textile applications [19,23]. Among them, polydimethylsiloxane (PDMS) [24], thermoplastic polyurethane (PU) [25] and rubber [26] are the most investigated ones.

The electrical conductivity of ECPCs is greatly affected by the concentration of the filler. A conductive network can be established across the polymer matrix when a minimum volume fraction of the conductive filler in the polymer is reached, known as the conductive percolation threshold. At the percolation threshold, the polymer composite, which was initially insulating material, eventually becomes conductive. Electrical conductivity increases exponentially with the filler concentration until a plateau is reached. The percolating behaviour is based on percolation theory, which Broadbent and Hammersley initially published in 1957 [27]. Two main mechanisms have generally been proposed to explained this behaviour: the tunnelling effect, and Ohmic conductance [19]. The first mechanism occurs when the concentration of added filler is low, which means that there is a dielectric layer between particles. Quantum physics demonstrates that even at low concentrations, certain electrons of the particles can jump and contribute to electrical conductivity in the system, which is known as the tunnelling effect. By adding more filler to the composite to reach the percolation threshold, the distance between particles decreases and adjacent fillers can make a pathway to pass free electrons. Here, the dominant conductive mechanism is Ohmic conductance. The transition from first mechanism to second is termed Percolation Theory, describing overall electrical conductivity in ECPCs.

Apart from filler concentration, other factors also affect the electrical conductivity of the composite, such as shape, size, orientation, aspect ratio and distribution of filler, nature of the polymer, the interaction between the matrix and the filler, etc. [28]. Based on the volume fraction of the filler, several analytical and mathematical models have been presented to predict the composite electrical conductivity and the percolation threshold [29,30]. Numerical simulations have also been proposed to study the electrical conductivity of polymer-based composites [31]. These methods compute the electrical property of simulated composites in a wide range of scales, for example, in the molecular scale using molecular dynamics [32] and Monte Carlo methods [33], in the microscale level using Brownian dynamics [34] and dissipative particle dynamics [35], and the macroscale scale using micromechanics [36] and finite element (FE) methods [37]. Many of these simulations are based on a representative volume element (RVE) model, defined as the smallest volume of the material that provides effective composite characteristics. The RVE model relies on the finite sample geometry for randomly distributed fillers [38]. Recently, several studies have reported the predicted electrical properties of composites based on RVE models combined with FE calculations. For instance, Tamayo-Vegas et al. [39] simulated the conductive behaviour of CNT-epoxy resin nanocomposite using the FE method. They found a good agreement between experimental and simulation result. Likewise, Tserpes et al. [40] studied the electrical performance of a bio-based composite comprised of carbon fiber fabric-epoxy resin using FE-RVE model. In conclusion, numerical simulations are a low-cost, robust and reliable technique for understanding the electrical properties of polymer composites using parametric data [31].

In this context, the main purpose of this study was to use a combination of experimental measurements, an analytical model, and computational simulation to investigate the conductivity behaviour under the pressure of polyurethane filled with micro-copper at various concentrations. Many studies in the literature have investigated the electrical properties of polymer composite filled with copper microparticles. Yaman and Taga [41] prepared unsaturated polyester composites with dendrite-shaped copper by in situ polymerization. They examined the influence of copper particle size (from 25 μm to 120 μm) and their relative concentration on the electrical conductivity of the polymer. They found that the conductivity increased with increasing concentration and particle size, reaching a maximum of 10^−2^ S∙cm^−1^ at about 55 vol.% of copper and the percolation threshold was at around 20 vol.% of copper. Similarly, Luyt et al. [42] synthesized low-density polyethylene (LDPE) and linear low-density polyethylene (LLDPE) with varying micro-copper sizes (>38 μm) using the melt mixing method. They found similar results to Yaman and Taga [41] with a percolation threshold of about 19 vol.%. Poblete et al. [43] reported the fabrication of PMMA (polymethylmethacrylate) filled with micro and nanoparticles of copper at different concentrations using hot compression moulding. They found the same tendency in the conductivity values with varying concentration and size, but the percolation thresholds, with the values of 9.28 vol.% for the micro-copper (3.25–4.75 μm), were lower than others reported in the literature. Low values of percolation threshold were found by Misiura et al. [44] and Mamunya et al. [45] for epoxy resin composite with copper obtained by in situ polymerization, by Mamunya et al. [46] for polyethylene filled with different types and content of carbon particles, and by Boudenne et al. [47] for polypropylene matrix filled with copper particles. Mamunya et al. [46] and Boudenne et al. [47] obtained the polymer composite by hot compression method. The low values of percolation threshold were due to the formation of a fractal structure of the composite that forms a conductive pathway at a lower concentration [46]. Mamunya et al. [46] also found that the composites exhibited high electrical conductivity, as well as good EMI shielding properties. However, none of these studies employed PU as a matrix. PU is more flexible and deformable than the other polymers used in the literature. Since the conductive behaviour depends on the nature of the polymer and the interaction between the matrix and the filler, as it was mentioned before, it is critical to explore the conductive behaviour for copper micro-additive/polyurethane (Cu-PU) composites. Furthermore, the effect of applied pressure on the electrical conductivity and percolation thresholds for polyurethane filled with different concentrations of micro-copper particles has never been investigated before. It was expected that changes in conductivity under pressure could be significant due to the stretchability of PU [48]. All the mentioned publications used analytical or mathematical models to describe the electrical properties of the composite. These analytical approaches can be used to obtain the electrical properties of the composite such as PT concentration, reducing cost and time of experimental measurements. In this work, analytical model based on the percolation thresholds theory developed by Krupa and Chodák [30] has been used to describe the electrical properties of our composite. On the other hand, computational simulations for studying the electrical properties of polymer micro-composite are extremely scarce. These simulations can predict the conductivity behaviour of a composite by knowing only few parameters, e.g., particle sizes, concentrations, and properties of each component. As a result, a simulation does not need event experimentation to determine the conductivity values.

In this study, a solution mixing method that usually provides a good filler dispersion was used to prepare the polyurethane with different concentrations of copper. The solution of PU and copper was spin-coated to produce the film membrane. The distribution of copper in the polymer was characterized using an optical microscope. The electrical conductivity of micro composites for different concentrations of copper under different pressures was measured using a developed setup. The experimental data was compared with the analytical model and FE-RVE simulation. Digimat-FE and Matlab R2021b softwares were used to perform the FE predictions and the fitting of the analytical model, respectively.

## 2. Materials and Methods

### 2.1. Materials

The thermoplastic polyurethane pellets under trademark, Elastollan^®^ 1180 A 10 000, BASF, were supplied by BASF plc, Cheadle, UK. Powder spheroidal copper microparticles with a size distribution from 10 to 25 μm, >98% purity, was purchased from Sigma-Aldrich, Dorset, UK. *N,N*-dimethylformamide (DMF, anhydrous, >99.8%) was obtained from Merck Company, Dorset, UK. All the chemicals were used as received, without further purification.

### 2.2. Preparation of Micro-Copper Polyurethane Films

The micro-copper polyurethane (Cu-PU) films with different concentrations of copper (0, 0.55, 1.2, 2.6, 4.3, 6.6, 9.5, 14 and 20 vol.%) were prepared using the solution mixing method followed by the spin-coating technique. Table 1 shows the concentration of copper in PU expressed as percent in volume (vol.%) and percent in weight (wt.%). About 9 g of DMF was added to 1 g of polyurethane in a 20 mL beaker. Then, using a hot plate, the mixture was stirred at 200 rpm and heated at 60 °C for about 4 h. These conditions were required for complete dissolution of the PU in DMF. After that, the mixture was again weighted to calculate the exact concentration of PU in DMF, it was about 10 wt.%. Following that, an appropriate amount of micro-copper was added into a portion of PU/DMF solution and then, the Cu/PU/DMF solution was continually stirred at 60 °C until a homogenous mixture was formed. During stirring, the beaker’s top was covered with aluminium foil to reduce the loss of DMF and to keep the viscosity of the solution constant, avoiding agglomeration of the PU and copper. Then, immediately and while the solution was still stirring, 0.3 mL of the mixture was deposited onto a square Teflon substrate (2.5 cm × 2.5 cm) that was previously placed in a spin coater from Ossila. After that, the sample was spun at 1000 rpm for 15 s. After the spin coater stopped, and coated substrate was dried at ambient temperature for 24 h in a fume hood. Once the substrate was dried, the Cu-PU film was removed from the Teflon substrate and the film’s thickness was measured using a digital calliper. The composite was ready for the resistance measurements. It should be noted that all weights in this method were performed using an analytical balance (±0.0001 g).

### 2.3. Characterization

The size and distribution of the micro-Cu powder was obtained using a scanning electron microscope (SEM, Tescan MIRA 3, TESCAN, Kohoutovice, Czech Republic). Before SEM characterization, a layer of conductive carbon was added on the sample’s holder to make sure the conductivity between the powders and the holder was established. The morphology and dispersion of micro-Cu powders in polyurethane was evaluated by optical microscopy at 20× magnification (Alicona^®^ InfiniteFocus Optical Microscope, BRUKER alicona, Bregenz, Austria). It is worth noting that the images were taken before removing the Cu-PU film composites from the Teflon substrates.

**Figure 1:**
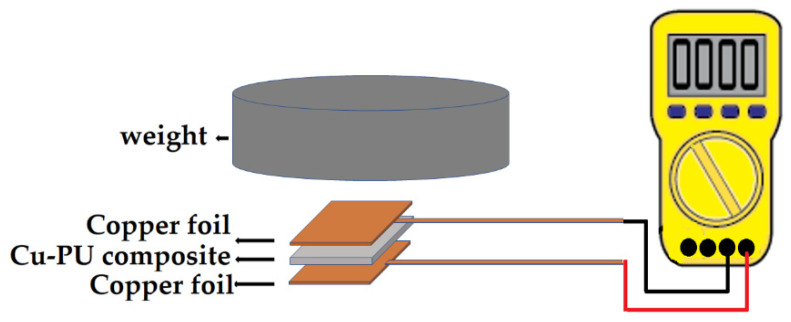
Schematic set up for resistance measurement.

Initially, the resistances were measured along the in-plane direction on the film (along the y and x direction) using a multimeter (UT58A, UNI-T, Dongguan, China) in the resistance mode. This was accomplished by spacing the two multimeter probes 1 cm apart on top the composite film. Unfortunately, the resistance values were over the multimeter scale, thus suggesting that the Cu-PU composites were non-conductive in the plane direction. Then, electrical resistance of the Cu-PU film composites under various pressure was measured for through-thickness (z direction) using the multimeter. The following approach was used. First, two copper foil tapes were pasted on the bottom and top sides of the Cu-PU films (0.5 cm × 1 cm). Second, an insulated stainless steel calibrated weight was put on top of the copper foil. Third, the electrical resistance was measured using the multimeter in resistance mode by touching the top and bottom of the copper foil with the two probes of the multimeter. The measurements were repeated three times and the averages and standard deviations were reported. The procedure was performed for various calibration weights: 5, 10, 20, 50, 75, and 100 g, that corresponded to 1, 2, 4, 10, 15 and 20 kPa pressure, respectively. It should be noted that the composite did not exhibited conductivity in the z-direction without pressure. The setup is schematically shown in Figure 1. Then, the volumetric resistivity was calculated using the measured resistance and following the Equation (1):

(1) $$\rho =R\cdot \frac{w\cdot l}{d}$$

where *ρ* is volumetric resistivity (Ω·m), *R* is the electrical resistance (Ω), w is the width (m), *l* is length (m) and *d* is the thickness (m) of the Cu-Pu films. Then, σ is the conductivity (S·m^−1^) obtained using the inverse of the volumetric resistivity, given by Equation (2):

(2) $$\sigma =\frac{1}{\rho }$$

### 2.4. Simulation

The finite element method (FE) was implemented by the MSC Software Digimat-FE (Hexagon, Munich, Germany) to compute the electrical performance of the micro-Cu-PU composites [39]. The software applied the constituent Ohm’s law to calculate the electrical conductivity of composites based on their 3D RVEs that composed of the micro-Cu particles in PU as a matrix. These 3D RVEs were created with the following parameters: (a) Cu particles were spherical with the diameter of 15 μm; (b) particles were distributed randomly in the 3D geometries; (c) relative distance between particles was allowed to be minimal; and (d) the RVEs had the same volume of 2.3 × 10^3^ μm^3^ for all simulations.

**Figure 2:**
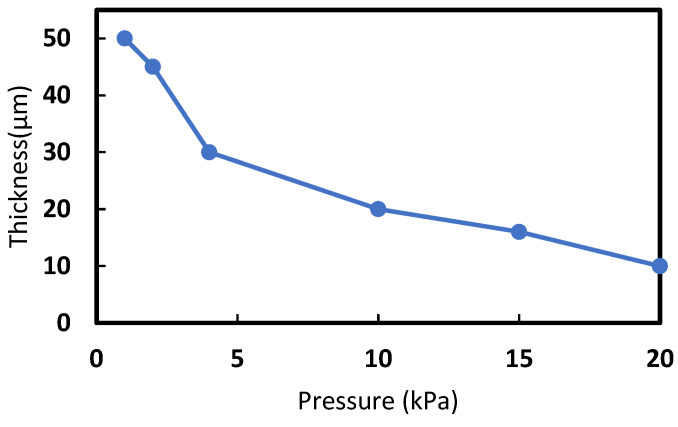
Evolution of the thickness of simulated composites for different applied pressures on the polymer composite.

**Figure 3:**
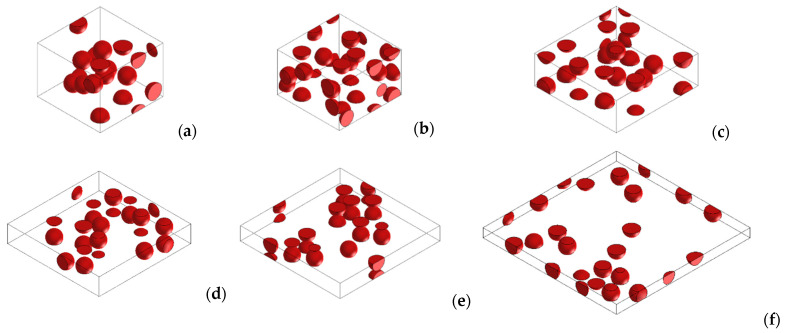
Simulated RVEs for 9.5. vol% Cu-PU composite for several applied pressures on the polymer composite. (**a**) 1 kPa, (**b**) 2 kPa, (**c**) 4 kPa, (**d**) 10 kPa, (**e**) 15 kPa and (**f**) 20 kPa.

**Figure 4:**
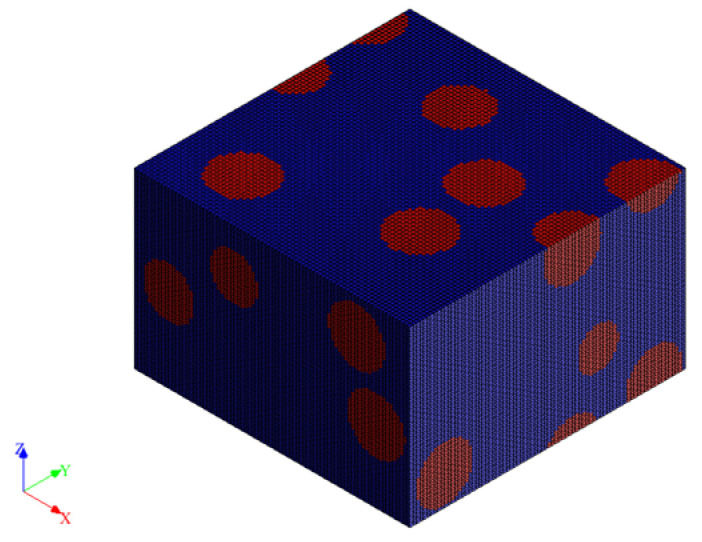
A meshed RVE for 9.5 vol.% Cu and 45 μm thickness for the Cu-PU film composite. The red dots are the Cu particles and dark blue is PU matrix.

In our simulations, the influence of the copper concentration and the effect of applying different pressures on the conductivity of the Cu-PU composite were studied simultaneously. In this regard, a series of 3D RVE was generated for the different Cu volume fractions (from 0 to 19.7 vol.%). Then, the effect of pressures on the composite conductivity was assumed to be caused by the diminution in the thickness. The pressures of 1, 2, 4, 10, 15, and 20 kPa were associated with the composite thicknesses of 50, 45, 30, 20, 16, and 10 μm, respectively. This was based on the thickness measured using a calliper and the mechanical properties of PU reported in the literature [48]. Figure 2 shows the thickness of composites for different pressures. The 3D RVEs for a specific copper concentration kept the number of Cu particles constant under the different loads in each set of simulations. For example, Figure 3 shows various RVEs representing different simulated microstructures for 9.5 vol.% of Cu for the several pressures on the composite. The RVE mesh generation with a number of non-conforming voxels converged with 512,000 elements, and it was kept the same for all the simulations. Figure 4 shows one of the meshed RVEs for the composite (9.5 vol.% Cu and 45 μm thickness). The conductivity was simulated in the Z direction (G_33_) that represented through-thickness conductivity of the Cu-PU composite film. The physical properties of copper and PU also required in the simulation are given in Table 2. It should be noted that the minimum conductivity that can be set up on the Digimat-FE is 1 × 10^−9^ S∙m^−1^, so that value was assumed to be the conductivity for PU matrix in the simulation calculations.

## 3. Results and Discussion

### 3.1. Characterization and Electrical Conductivity

**Figure 5:**
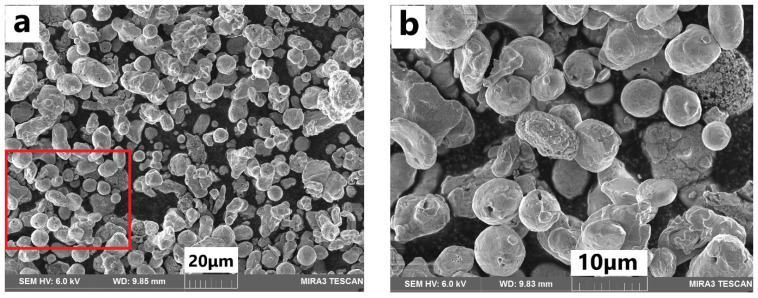
SEM image of the Cu particles in the as-received condition: (**a**) general image; (**b**) magnified image of the red square in (**a**).

**Figure 6:**
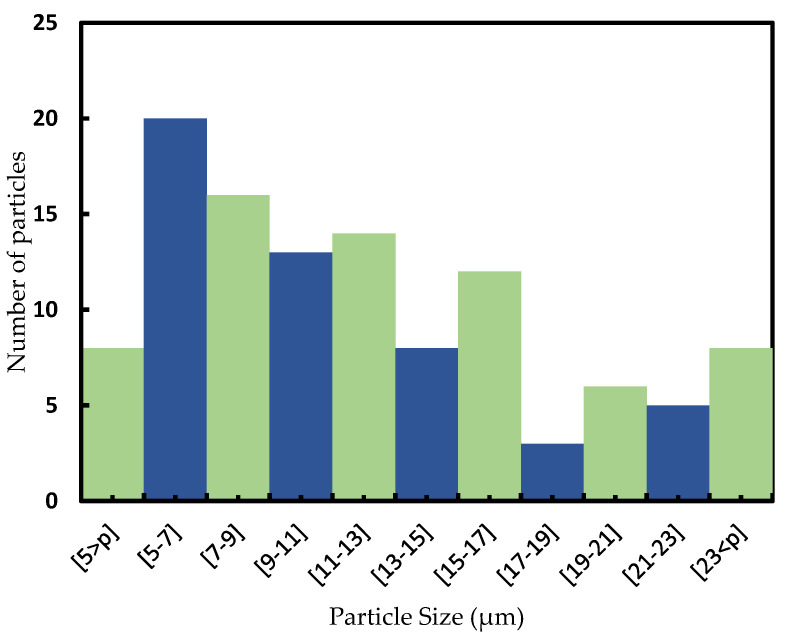
Number of particles vs particle size histogram showing the distribution of Cu powder. The numbers in brackets on the x-axis denotes the size range in microns. The particle size distribution was based on the image evaluation of 113 particles.

Figure 5 shows the SEM images of the Cu particles in as-received condition. The general image (Figure 5a) shows that the size and morphology of the particles was very similar and thus confirms the homogeneity of the powder. To characterize the particles in more detail, a magnified image is shown in Figure 5b. It was observed that the size of the particles ranged from about 5 to 25 μm and most of them were not perfectly round but exhibited some irregularities such as spiky particles with sharp protrusions. Figure 6 shows particle size distribution of Cu powder, obtained by using ImageJ software. As it is shown in Figure 6, the particle size was in the range of 5–23 μm, mainly, and the mean size was about 12 μm.

**Figure 7:**
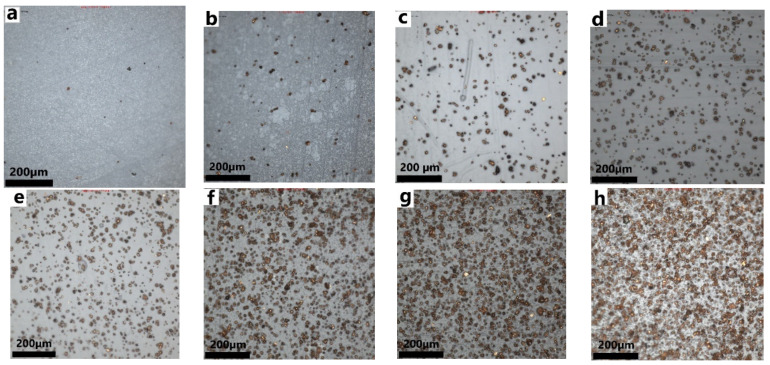
Optical microscopy images showing different content of Cu particles dispersed in PU: (**a**) 0.55 vol.%, (**b**) 1.2 vol.%, (**c**) 2.6 vol.%, (**d**) 4.3 vol.%, (**e**) 6.6 vol.%, (**f**) 9.5 vol.%, (**g**) 14 vol.% and (**h**) 20 vol.%.

Particles of Cu were added to PU in different concentrations, from 0.55 vol.% to 20 vol.%, thus forming different Cu-PU composites as shown in the optical microscopy (OM) images of Figure 7. The distribution of particles across the composites was very homogeneous, although with some slight local agglomeration for concentrations of 6.6 vol.% and higher, which was similar to the observation from other authors [41,42]. As the concentration of Cu particles increased from 0.55 vol.% (Figure 7a) to 20 vol.% (Figure 7h), the relative distance between particles decreased, from around 200 μm to 4 μm, respectively. This could indicate that the density of Cu in the composites witnessed an increment proportionate to the higher filler content.

The fillers were made out of pure copper (purity 98%) and therefore they were electrically conductive (~5.96 × 10^7^ S/m) [20]. Although increasing the conductive filler loading resulted in a reduction in the distance between particles (Figure 7), this vicinity of fillers in the composites did not provide a continuous electron pathway in the in-plane direction. This was experimentally found from measuring the conductivity of samples in the in-plane direction since they were non-conductive. It was reported by Schmidt et al. [50] that polymer’s tendency to cover the fillers produced a dielectric layer around the particles. In addition, despite some very local particle agglomeration, normally less than 10 particles, especially for concentrations higher than 6.6 vol.%, the composite did not exhibit conductivity in the in-plane direction due to the presence of an insulator PU layer around the clusters. This aggregation in the composites prepared using the spin-coating technique was described by Schmidt et al. [50]. They termed this the carpet sweeper mechanism. They mentioned that the developed velocity gradient across the composite when using spin-coating to fabricate the composite was associated with the agglomeration. Putson et al. [51] also reported that the clustering was due to the viscous nature of the PU matrix and surface energy of Cu particles. In the literature, other authors have found that micro-copper composites were conductive along the in-plane direction [41,42], but they used a much higher concentration of copper than we did in this study. However, as the composites have 3D structures, they showed electrical conductivity in the through-thickness (z direction); this might be related to the clustering in the z direction.

To better understand how the proximity between particles affected the electrical conductivity, different pressures were applied to the composites since it was expected that a pressure increase should promote particles to get closer to each other.

**Figure 8:**
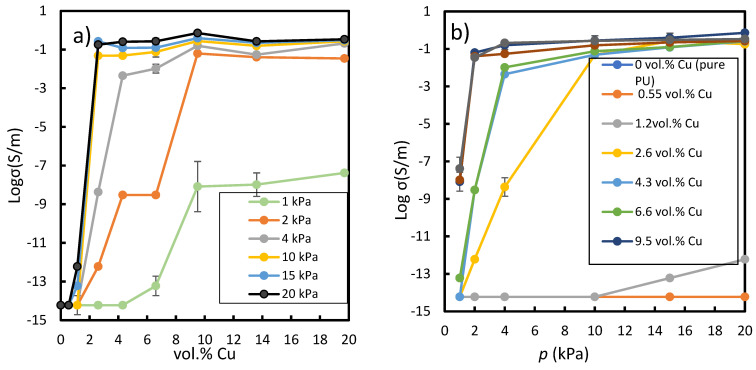
(**a**) Electrical conductivity vs. concentration of Cu for the different pressures (1–20 kPa), and (**b**) electrical conductivity vs. applied pressure for different concentrations of Cu particle in volumetric percent. In b), it should be noted the dark blue line-symbols for 0 vol % Cu is overlapped with orange line-symbols for 0.55 vol % Cu.

Figure 8a shows the correlation between Cu concentration and electrical conductivity in the composites when different pressures were applied. In general, it was observed that for small concentrations of copper, the electrical conductivity was very small and approximately constant and close to that of the polymer matrix. There was a certain concentration of particles beyond which the conductivity increased rapidly (percolation threshold) and reached a maximum conductivity. This conductivity level remained almost constant regardless of the increase in copper concentration. It was observed that the percolation threshold shifted to lower copper concentrations, i.e., 1.4 vol.%, as the applied pressure increased. A similar transition of percolation threshold was reported by Bloor et al. [52] when investigating the effect of different deformations on the electrical properties of a nickel composite with the particle size between 3.5 and 4.5 μm. Before that, in 1998, Chelidze and Gueguen [53] termed this change of percolation as pressure-induced percolation transition (PIPT). For 10 kPa and higher, the percolation threshold reached a minimum value of about 1.4 vol.% and remained practically the same with increasing pressure. Moreover, the slope of the conductivity as a function of the concentration of copper got steeper with increasing pressure up to 10 kPa, and remained basically the same for higher applied pressure, i.e., 15 and 20 kPa.

It was observed that the lowest conductivity for all concentrations were obtained when the lowest pressure, 1 kPa, was applied. In this case, the conductivity surged up after 6.5 vol.% (percolation threshold) and reached a maximum of around 8 × 10^−9^ S·m^−1^. For 2 kPa, the same trend was observed but the percolation threshold was about 5.5 vol.% Cu and a maximum conductivity of about 6 × 10^−2^ S∙m^−1^ was reached. The threshold concentration in copper decreased to about 2.1 vol.% for 4 kPa and a similar maximum conductivity was reached under lower pressure but with a smaller copper concentration range, so the conductivity as a function of Cu vol.% exhibited a steeper slope. For 10 kPa and beyond, the vol.% Cu percolation threshold was very similar, about 1.4 vol.% copper and a maximum conductivity of 7.2 × 10^−1^ S·m^−1^ was reached. In general, for all the applied pressures, the increase in Cu concentration in the PU increased the conductivity of the composite [41,43,47,54,55]. This was because the probability of percolation increased as the conductive filler content rose [10,56].

Figure 8b shows the conductivity of composites as a function of applied pressure up to 20 kPa. Composites with 0.55 and 1.2 vol.% fillers exhibited an electrical behaviour about the same as for pure PU (dark blue line, Figure 8b), which overlapped with the orange line for 0.55 vol.%. No significant change in the electrical conductivity was observed even for 15 kPa, indicating that they were non-conductive. However, for concentrations in copper as high as 2.6 vol.%, the slope steepness of the electrical conductivity as a function of applied pressure increased dramatically, indicating high sensitivity to the external applied pressure. The electrical conductivity of 2.6 vol.% Cu-PU composite increased progressively when applied pressure increased, from non-conductive to conductive composite, 10^−14^ to 1.8 × 10^−1^ S∙m^−1^ for 1 kPa and 20 kPa, respectively. The gradual increase in the conductivity of 2.6 vol.% Cu-PU composite (Figure 8b yellow line) with the applied pressures characterized a highly sensitive composite under different pressures, making this composite a reasonable candidate for many applications such as switches and tactile sensors [12]. However, for pressures higher than 4 kPa, greater Cu concentrations than 9.5 vol.% did not have dramatic changes in conductivity. This means that the high-concentration composites did not show such electrical sensitivity to the external pressure after a critical particle concentration point. Composites with 9.5, 14, and 20 vol.% filler followed a similar trend to the external pressure applied in the z direction, increasing the conductivity as the pressure increased. In conclusion, the conductivity of Cu-PU composites with particle concentrations more than 2.6 vol.% increased as the pressure rose. The effect of pressure on the conductivity may be related to a decrease in the thickness of the film as the pressure increases, resulting in a decrease in the distance between Cu particles and hence the creation of a conductive network.

### 3.2. Analytical Model

In this work, the electrical properties of Cu-PU composites were described using an analytical method developed by Krupa and Chodák [30]. This model was successfully used by Boudenne et al. to estimate Cu-polypropylene conductivity [47]. The equation of the model is an exponential function of the filler volume fraction, and it is described as follows:

(3) $$Log\left(\frac{\sigma_{c}}{\sigma_{m}}\right)=B{\left(1-e^{-\alpha \phi }\right)}^{n}$$

where $\sigma_{c}$ and $\sigma_{m}$ are the electrical conductivity of composite and matrix, respectively. Here, ϕ is the volume fraction of filler. *B*, *α* and *n* are fitting parameters. The percolation concentration, $\phi_{c}$ , is defined by the inflexion point $\phi_{i}$ and is given by the Equation (4).

(4) $$\phi_{i}\equiv \phi_{c}=\frac{\ln\left(n\right)\;}{\alpha }$$

**Figure 9:**
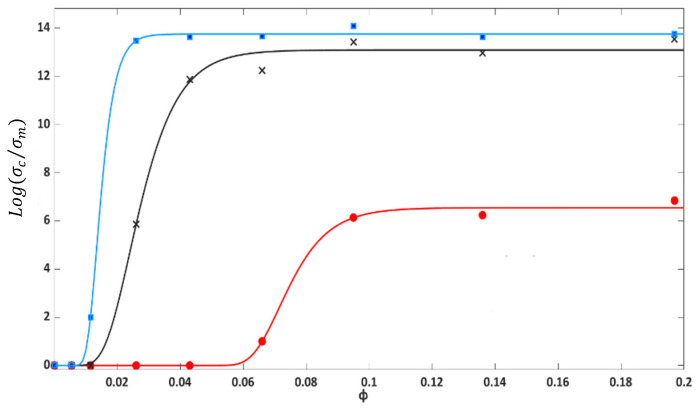
Relative electrical conductivity ( $\frac{\sigma_{c}}{\sigma_{m}}$ ) of Cu-PU composites under pressure: 1 kPa (red), 4 kPa (black) and 20 kPa (blue). The symbols represent the experimental conductivity and curves are the values from Equation (3) using the parameters of Table 3.

The parameter B may be approximated as:

(5) $$B≅\log(\sigma_{c,max}/\sigma_{m})$$

where $\phi_{c,max}$ is the maximum value of electrical conductivity of composites at the highest experimental concentration of the filler. In this work, this model was fitted to the experimental conductivity for different pressures using Matlab R2021b. Table 3 gives the percolation concentrations, $\phi_{c}$ and the fitted parameters *B*, *a*, and *n* of the Equation (3) for PU-Cu composites under various pressures. To simplify the graph, Figure 9 shows the experimental and calculated relative conductivity for three applied pressures 1 kPa, 4 kPa, and 20 kPa, that represented the lowest, middle, and maximum pressure, respectively. The results showed that the model fitted the data well, with a standard deviation, s, of about 0.34 and could also correctly predict the percolation concentration at various pressures. As can be seen in Figure 9 and Table 3, the percolation concentration for PU-Cu composite ranged from 7.1 vol.% to 1.4 vol.% and decreased with the pressure rise. No substantial change was seen in the $\phi_{c}$ at pressure above 15 kPa. The data clearly showed the phenomenon as PIPT. The maximum of conductivity, *σ_c,max_*_,_ was also calculated using Equation (5) and is given in Table 3. The *σ_c,max_* increased with the rise in pressure, as seen in Table 3. Both *σ_c,max_* and $\phi_{c}$ _._ showed a jump in their values when the pressure changed from 1 kPa to 2 kPa. This means that the critical pressure at which the PU-Cu composite became conductive was 2 kPa.

### 3.3. Numerical Simulations

**Figure 10:**
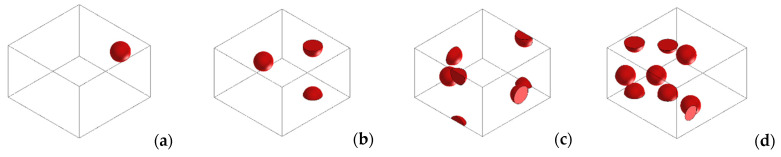

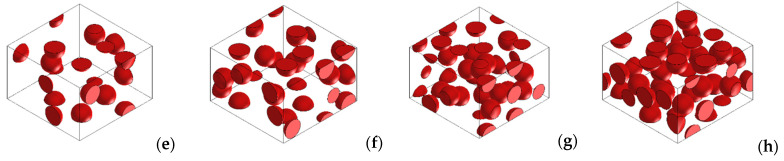
3D RVEs simulated geometries for 1 KPa applied pressure as function of Cu percentages in the PU: (**a**) 0.55 vol.%, (**b**) 1.2 vol.%, (**c**) 2.6 vol.%, (**d**) 4.3 vol.%, (**e**) 6.6 vol.%, (**f**) 9.5 vol.%, (**g**) 14 vol.% and (**h**) 20 vol.%.

In our simulations, we simultaneously studied the effect of copper concentration and the application of various pressures on the conductivity of Cu-PU composites. As mentioned, Figure 3 illustrates several RVEs for 9.5 vol.% Cu when different pressures were applied to the composite. The number of fillers for each concentration was the same in all the pressures since the volume of the generated 3D RVEs (2.3 × 10^3^ μm^3^) were independent of applied pressure (see Figure 3). This makes the comparison between simulations more feasible. Another factor that affects the conductivity is the filler concentration. When the concentration of filler increased, more volume was occupied by fillers, resulting in an increased probability of reaching the percolation threshold [39,56]. The concentration effect on the simulation is shown in Figure 10, which presents the simulated 3D RVEs geometries of the composites as a function of Cu content when 1 kPa pressure was applied on the composite. The same procedure was used for all the pressures.

**Figure 11:**
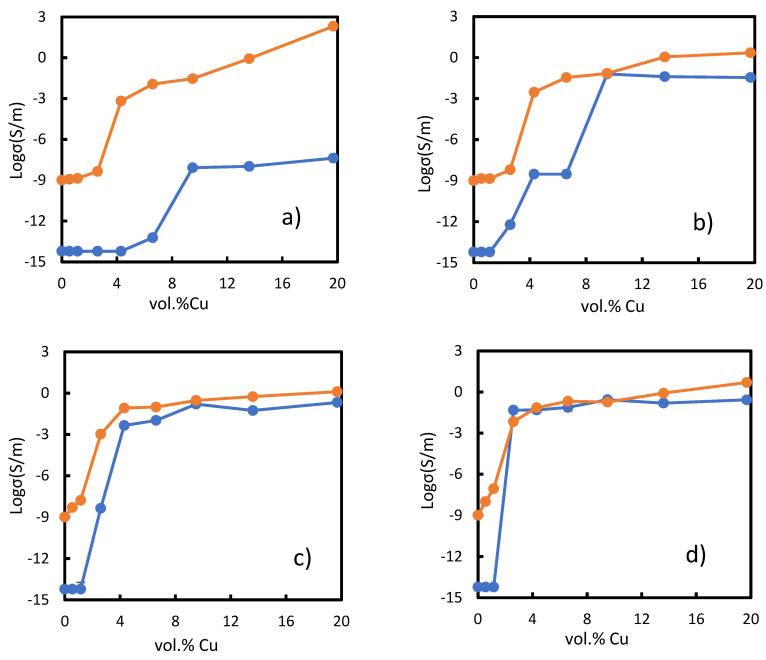

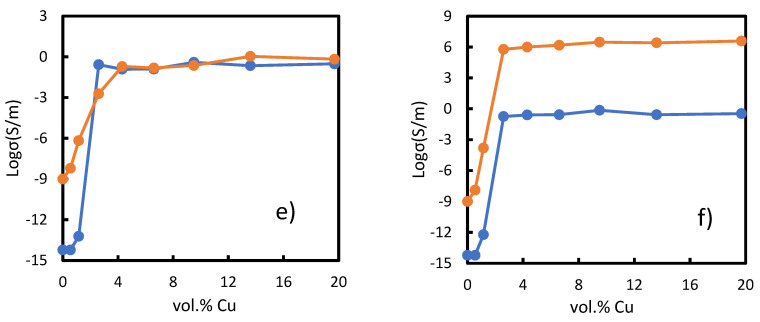
Comparison between the electrical conductivity of experimental and simulation results affected by applied pressures: (**a**) 1 kPa, (**b**) 2 kPa, (**c**) 4 kPa, (**d**) 10 kPa, (**e**) 15 kPa and (**f**) 20 kPa. The orange curve-points represent the simulated conductivity, and the blue curve-points represent our experimental conductivity.

Figure 11 simultaneously shows the conductivity of the composites obtained by the experiments and the corresponding simulated ones. Most of the experimental results agreed with simulated ones, except the lowest and highest amount of applied pressure, 1 kPa and 20 kPa, (Figure 11a,f), for which the simulation results showed a conductivity of 10^4^ S∙m^−1^ higher than the experimental results. Before the percolation threshold for all the results, there was about 10^4^ S∙m^−1^ difference in conductivity between the simulation and experimental values. This might be associated with software limitations, since the lowest accepted value was 10^−9^ S∙m^−1^ and the real conductivity for pure PU is 10^−14^ S∙m^−1^ [39].

The percolation threshold has been defined as a specific concentration for which further addition of that amount of filler makes the conductivity of a composite witness a significant increment. Figure 11 also shows the dependency of the percolation threshold upon applied pressure. As can be seen in the figure, our numerical method can predict the PIPT phenomenon described by Chelidze and Gueguen [53] and measured experimentally by Bloor [52]. Applying the two lowest pressures, 1 and 2 kPa, the simulated results showed the percolation threshold for a concentration about 2.6 vol.%. These values were lower than the experimental ones. The simulated percolation threshold for higher applied pressures occurred at concentrations of about 1.2 vol.% or lower. In general, the simulated results correctly predicted the experimental percolation threshold for all the pressures, except for 1 and 2 kPa.

An increase in conductivity as the Cu concentration and pressure on the composites increased was observed from the simulations and experiments. In this regard, the maximum conductivity of the simulated results was achieved when 20 kPa pressure was applied on the composites, and this remained almost constant for concentrations higher than 2.6 vol.% copper (see Figure 11f). The maximum value predicted from simulations was 3.79 × 10^6^ S∙m^−1^ for the composite with 20 vol.% Cu. This highest conductivity was close to the Cu powder conductivity. Figure 3f, which corresponds to the largest pressure, 20 kPa, and the lowest thickness, shows that copper particles went through the composite thickness. This might explain why the conductivity was comparable to that of pure copper. However, the experimental maximum value was lower than the simulated one. This might be attributed to the fact that the real size of the copper particles ranged from about 5 to 25 μm and in the simulation it was a set to be 15 μm. Higher values of conductivity at higher concentrations is due to the fact that filler volume concentration surpasses the percolation threshold, and therefore the electrons can be transmitted through direct contact of the metallic particles [45]. This is termed as “metallic conductivity”. These conditions make the electron transportation the same as when no insulator matrix is presented (pure Cu). On the other hand, the conductivity for the lower concentration of copper is controlled by the tunnelling effect, in which some electrons can be transferred between the metal particles insulated with the layer of polymer [19].

To summarize, as previously commented, the software had the limitation of not allowing the input of the real conductivity value for PU, in this case 10^−14^ S∙m^−1^. This had an effect on the differences observed between the experimental and simulation results, around five orders of magnitude before the PT concentration [39]. Despite this software limitation, conductivity of the composites obtained by experiments and simulations for concentrations higher than the percolation threshold were closer to each other. Overall, the numerical approach could predict the general behaviour of the conductivity as a function of Cu concentration. This indicated that the model can describe both mechanisms: the tunnelling effect, and Ohmic conductance.

## 4. Conclusions

In this study, micro-size Cu particles (less than 25 μm) were incorporated into PU using a solution mixing method followed by a spin-coating technique to fabricate composite films with particle concentrations ranging 0.55 to 20 vol.% Cu. The optical microscopy images showed that the Cu particles were homogeneously distributed across the composites. While the electrical behaviour of these composites under pressure were investigated experimentally, a semi-analytical method and a numerical simulation technique using the FE method based on the RVE model were implemented to compute the electrical properties of the composites. In this study, it was observed that changes in conductivity only occurred in the z-direction under pressure (1 to 20 kPa). For PU composites with particle concentrations higher than 2.6 vol.%, the conductivity of the composites increased as the pressure increased. The effect of the pressure on the conductivity of these composites could be attributed to the reduction in their thickness as the pressure rises, which results in a decrease in the distance between Cu particles and therefore in the formation of a conductive network. At constant pressure, the conductivity increased with the increase in Cu content in PU. The maximum value obtained experimentally was about 7.2∙10^−1^ S∙m^−1^ for a composite with 20 vol.% at 20 kPa.

The accurate values of the percolation threshold (PT) concentration were obtained using the semi-analytical model reported by Krupa and Chodák [30]. The PT concentration for the PU-Cu composite ranged from 7.1 vol.% to 1.4 vol.%, and decreased as the pressure rose. Regarding the simulations, our study revealed that FE-RVE simulation accurately predicted the PT concentration and offered a good representation of the experimental conductivity after the PT. The following findings were derived from this research:

(a) The obtained Cu-PU composite films could be used as a basic material for flip-chips, switching devices, and tactile sensors, as they are flexible, easy to prepare, lightweight, conductive, and their conductivity can change from 10^−14^ to 3.5 10^−1^ S·m^−1^ depending on the applied pressure (1 to 20 kPa);
(b) The FE-RVE simulation could be used to describe the change in the electrical conductivity of polymer–metal composites subjected to different pressures. This also includes the dominating electron transport mechanisms before and after the percolation threshold. This research could help to reduce the number of expensive and time-consuming experiments carried out. Further investigation would be required to find out more about the exhaustive coverage of this method for a wide range of composites, including carbonaceous–polymer, hybrid fillers and reinforced composites.

## Figures and Tables

**Table 1:** Concentration of copper in the polyurethane expressed in percent of weight (wt.%) and percent of volume (vol.%) used in Cu-PU film composite.

Concentration of Copper in PU								
wt.%	5	10	20	30	40	50	60	70
vol.%	0.55	1.2	2.6	4.3	6.6	9.5	14	20

**Table 2:** Physical properties of the components of Cu-PU film composite [49].

Material	Conductivity (S·m^−1^)	Density (Kg/m^3^)
PU	10^−14^	1110
Cu	5.97 × 10^7^	8940

**Table 3:** The values of adjustable parameters, *B*, *α*, *n* of Equation (3), $\phi_{c}$ , calculated percolation threshold concentration from Equation (4) in volume fraction and vol.% and *σ_c,max_* is the calculated maximum of conductivity. *P* is the pressure and *s* is the standard deviation of model.

*P* (kPa)	*B*	*α*	*n*	$\phi_{c}$	$\phi_{c}$ (vol.%)	*σ_c,max_* (S/m)	*s*
1	6.545	115.7	3899	0.0710	7.10	3.5∙10^−8^	0.17
2	13.15	71.89	14.77	0.0370	3.70	1.4∙10^−1^	0.73
4	13.09	117.7	16.74	0.0240	2.40	1.3∙10^−1^	0.39
10	13.35	371.1	528.3	0.0170	1.70	2.3∙10^−1^	0.27
15	13.55	723.1	9076	0.0126	1.26	3.5∙10^−1^	0.33
20	13.75	313.3	69.82	0.0135	1.35	5.6∙10^−1^	0.16
